# Effect of energy transfer on the optical properties of surface-passivated perovskite films with CdSe/ZnS quantum dots

**DOI:** 10.1038/s41598-019-54860-1

**Published:** 2019-12-05

**Authors:** Il-Wook Cho, Mee-Yi Ryu

**Affiliations:** Department of Physics, Kangwon National University, Chuncheon, 24341 Republic of Korea

**Keywords:** Materials science, Physics

## Abstract

Surface passivation is an effective method to protect the surfaces and improve the luminescence properties of perovskite (PS) films. CdSe/ZnS core-shell quantum dots (QDs) have been employed for surface passivation of PS films because of their size-dependent tunable bandgaps. Herein, the energy transfer (ET) behavior of CH_3_NH_3_PbI_2_Br PS films covered with CdSe/ZnS QDs (QD/PS hybrid structures) is characterized by using photoluminescence (PL) and time-resolved PL spectroscopy. The PL decay time and the integrated PL intensity of the QD/PS hybrid structure increase compared with those of the bare PS films, owing to ET from the QDs to the PS and reduced charge traps. The ET efficiency increases from ~7% to 63% for the QD/PS hybrid structure when the core diameter of the QDs decreases from 6.5 to 2.7 nm, respectively. This can be explained by the charge transfer rate enhancement due to the control of energy level alignment of QDs. These results allow us to understand fundamental mechanisms such as ET from QDs to PS films as a function of the size of the QD.

## Introduction

Methylammonium lead halide perovskites (CH_3_NH_3_PbX_3_, where X = Cl^−^, Br^−^, or I^−^) are of great interest in optoelectronic applications because they offer many advantages such as a tunable bandgap^1,2^, high absorption coefficient^3^, high electron/hole mobility^4^, and low-cost solution processing. In addition, the power-conversion efficiencies achieved by solution-processed perovskite (PS) solar cells are comparable to those of silicon and semiconductor thin film devices^5,6^. However, solution-processed PS contains many charge traps at the grain boundaries and on the surface, leading to increased non-radiative recombination^7–9^ and instability of the PS film in air due to humidity. These issues need to be resolved for the successful application of such materials. Therefore, suppressing these defect states is crucial to the development of PS-based devices to ensure their superior performance and long-term stability.

Surface passivation is a common method for minimizing charge traps induced by structural defects and for protecting the surface from the moisture in air. Several researchers have reported the effect of surface passivation on the optical and electrical properties of materials and demonstrated enhanced luminescence properties by suppressing non-radiative recombination and decreasing defect states^10–13^. However, elucidation of the fundamental and physical processes involved is necessary for optical properties to be similarly improved by surface passivation.

Therefore, in this study, we conducted an in-depth analysis of the surface passivation effect on the optical properties of PS films. In order to investigate the impact of surface passivation, we introduced the energy transfer (ET) phenomenon in surface-passivated PS films. The fluorescence resonance energy transfer is a non-radiative transfer process of excitation energy from a donor to an acceptor, which is affected by overlap between the emission and absorption spectra of the donor and acceptor, respectively. The charge transfer (CT) process in PS films covered with CdSe/ZnS core-shell quantum dots (QDs) (QD/PS hybrid structures) denotes the transport of photo-excited electrons and/or holes from a donor (QD) to an acceptor (PS)^14^. Additionally, the CT process has been demonstrated in coupled systems such as two-transition-metal dichalcogenides (TMDCs)^15,16^, organic–TMDCs^17^, and semiconductor–nanoparticle hybrid structures^18^. In particular, the ET process between a mixed halide PS (CH_3_NH_3_PbI_2_Br) and CdSe/ZnS core–shell quantum dots (QDs) has been investigated as a function of the core diameter of the QDs using photoluminescence (PL) and time-resolved PL (TRPL) spectroscopy in this work. We compared the PL decay behaviour and the integrated PL intensity of this system to those of bare PS and bare QD films and demonstrated an improved luminescence yield and an ET efficiency of 63% in the QD/PS hybrid structure for a QD of diameter 2.7 nm.

## Results and Discussion

**Figure 1:**
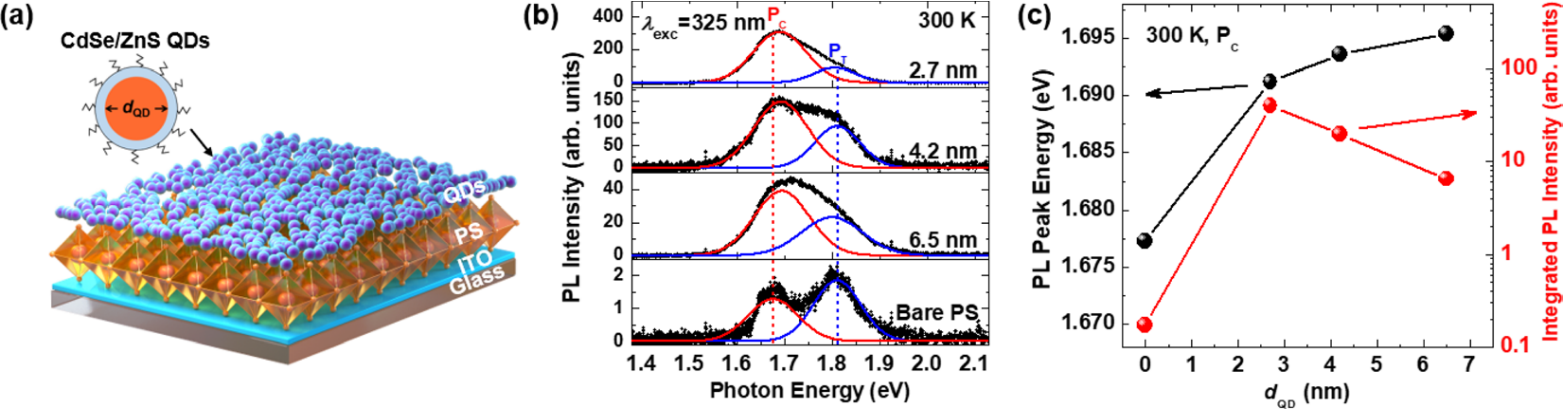
(**a**) Schematic illustration of the QD/PS hybrid structure and the CdSe/ZnS core-shell QDs. (**b**) PL spectra (black lines) for the bare PS and QD/PS hybrid structures. The PL peaks at P_C_ (red lines) and P_T_ (blue lines) were extracted using Gaussian fitting. (**c**) PL peak energies (black dots) and integrated PL intensities (red dots) taken at P_C_.

Figure 1(a) shows a schematic illustration of the QD/PS hybrid structure used in this study, consisting of PS covered with CdSe/ZnS core–shell QDs on indium tin oxide (ITO) coated glass substrates. In addition, the illustration of CdSe/ZnS QDs is shown. The thickness of the ZnS shell was 0.6 nm, and the diameters of the CdSe cores (*d*_QD_) were 2.7, 4.2, and 6.5 nm. In order to confirm the morphology, crystallinity, and absorbance of the QD/PS hybrid structures, we performed scanning electron microscopy (SEM), X-ray diffraction (XRD), and ultraviolet-visible (UV-vis) measurements (see Supplementary Information (SI), Figs. S1 and S2). The diffraction peaks at 14.3° and 28.8° in Fig. S2(a) were assigned to the (100) and (200) planes of the cubic phase of the PS, respectively^1^. The diffraction patterns and SEM images for the QD/PS hybrid structures were almost the same as those for the bare PS, indicating that covering with QDs did not significantly affect the crystallinity and morphology of the PS films. In addition, we acquired PL spectra of the bare QDs with various *d*_QD_ and plotted these along with the UV-vis absorption spectrum of ITO, bare PS/ITO, and QD/PS/ITO, as shown in Fig. S2(b). The UV-vis absorption spectrum for the bare PS exhibits an absorption edge at 1.78 eV, which matches well with an absorption edge reported for CH_3_NH_3_PbI_2_Br^1,19^. The PL peak energies of the bare QDs can be seen near 2.29, 2.06, and 1.90 eV for a *d*_QD_ of 2.7, 4.2, and 6.5 nm, respectively, which are higher than the bandgap of the bare PS.

In order to clarify the effect of QD surface passivation on the luminescence properties of the PS film, the PL spectra of the PS films covered with QDs were collected using a 325 nm excitation wavelength (*λ*_exc_) at 300 K, as shown in Fig. 1(b). The PL spectra of the PS films exhibited two emission peaks near 1.68 (P_C_) and 1.81 eV (P_T_), which could be attributed to the phase segregation between bromide-rich cubic and iodide-rich tetragonal phases induced by laser irradiation^20–23^. This light-induced phase segregation caused additional charge traps to form^23^. The PL peaks for P_T_ did not change even when the surface of PS was covered with QDs, while the PL peaks for P_C_ for the QD/PS hybrid structure showed a blueshift compared with that of the bare PS. In addition, the integrated PL intensity for P_C_ in the QD/PS hybrid structure became dominant upon covering the PS with QDs, which could be attributed to the prevention of light-induced phase segregation. Therefore, the discussion herein will focus on the changes in P_C_, because the P_C_ is the main PL peak, and PS has a cubic phase, as seen in Figs. 1(b) and S2(a) respectively. The PL peak energies and integrated PL intensities for P_C_ are shown in Fig. 1(c). The PL peak of the QD/PS hybrid structure showed a blueshift of approximately 14–18 meV compared with that of the bare PS. This P_C_ blueshift is attributed to the decreased number of charge traps due to QD surface passivation^11,24^. The integrated PL intensity of P_C_ for the QD/PS hybrid structure with *d*_QD_ of 2.7, 4.2, and 6.5 nm increased considerably to about 252, 123, and 33 times the value of the bare PS, respectively, as shown in Fig. 1(c). The increased PL intensity could be explained by increased radiative recombination resulting from the combined effect of reduced defect states and ET from the QDs to the PS layer, caused by QD surface passivation. The organic ligands of QDs can also passivate the PS surface and affect charge transfer. The organic ligands cause hole trapping at the QD surface, increasing both charge separation in QDs and consequently the rate of electron transfer^25^. The QD/PS hybrid structure with a *d*_QD_ of 2.7 nm exhibited the greatest PL enhancement, which will be discussed later.

**Figure 2:**
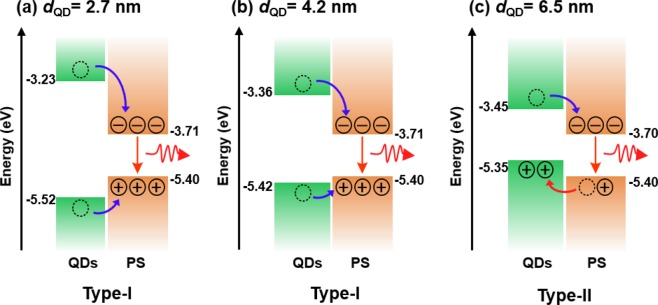
Energy level diagrams for the QD/PS hybrid structures with *d*_QD_ of (**a**) 2.7 nm, (**b**) 4.2 nm, and (**c**) 6.5 nm.

Figure 2(a–c) show the energy level diagrams for the QD/PS hybrid structure with a *d*_QD_ of 2.7, 4.2, and 6.5 nm, respectively. The valence band maximum (VBM) value for CdSe with various *d*_QD_ is given by *E*_VBM_(*d*_QD_) = *E*_VBM_(∞) − *Ad*_QD_^−*B*^, where *E*_VBM_(∞) = –5.23, *A* = 0.74, and *B* = 0.95^26^, and the VBM value (−5.40 eV) of the PS (CH_3_NH_3_PbI_2_Br) was derived from literature^27^. The conduction band minimum (CBM) values of the QDs and PS were determined using the PL peak energies for the QDs and the PS, respectively. The energy level diagrams of the QD/PS hybrid structure suggest that charge carrier transport could have occurred due to the energetic offset between the CBM and VBM of the QDs and PS. The QD/PS hybrid structures with *d*_QD_ of 2.7 and 4.2 nm showed type-I band alignment because the CBM of the QDs was located at a higher energy than that of the PS, and the VBM of the QDs was at a lower energy than that of the PS. Thus, in the case of the QD/PS hybrid structures with *d*_QD_ of 2.7 and 4.2 nm, both electrons and holes in the QDs were transferred to the PS layer. On the other hand, the QD/PS with a *d*_QD_ of 6.5 nm showed a type-II band alignment because the VBM of these QDs was located at a higher energy than that of the PS; thus, the electrons in the QDs could be moved to the PS layer, while the holes in the PS layer could be transferred to the QD layer, as shown in Fig. 2(c).

**Figure 3:**
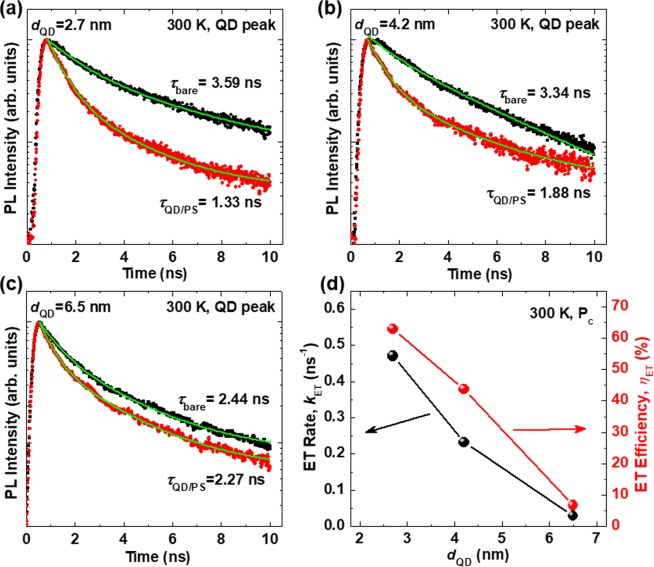
PL decay curves at QD peak for the bare QDs (black dots) and QD/PS hybrid structures (red dots) with *d*_QD_ of (**a**) 2.7 nm, (**b**) 4.2 nm, and (**c**) 6.5 nm. The green lines indicate approximated curves obtained by fitting with a bi-exponential function for the bare QDs and the QD/PS hybrid structures. (**d**) ET rate (black dots) and ET efficiency (red dots) of the QD/PS hybrid structures as a function of *d*_QD_.

In order to verify the ET process between QDs and PS, TRPL measurements were performed. Figure 3(a–c) display the PL decay curves recorded at the QD peak of the bare QDs and the hybrid structure at 300 K. The PL decay curves of the bare QDs and the hybrid structure were fitted well by a bi-exponential decay function, and the fast and slow decay times, *τ*_1_ and *τ*_2_, were estimated^14^. The average decay time (*τ*_ave_) was estimated using the relationship *τ*_ave_ = ∑*A*_i_*τ*_i_^2^/∑*A*_i_*τ*_i_. The detailed decay parameters and the calculated results are provided in the SI (Tables S1 and S2). The average PL decay times of the QDs in the QD/PS hybrid structure shortened to 1.33 (2.7 nm), 1.88 (4.2 nm), and 2.27 ns (6.5 nm) compared with the 3.59 (2.7 nm), 3.34 (4.2 nm), and 2.44 ns (6.5 nm) noted for the bare QDs, respectively. The PL decay times for the bare QDs were shorter than the values found in literature^28,29^. In addition, we obtained PL decay curves for solutions of the bare QDs (see SI, Fig. S3), and the decay time of the bare QDs in solution was longer than that of the bare QDs on ITO substrates. The relatively short decay time for the bare QDs on ITO substrates is attributed to exciton dissociation or exciton diffusion^30^. Using the equation *τ*_QD/PS_^−1^ = *τ*_bare_^−1^ + *τ*_ET_^−1^, we calculated the ET efficiency (*η*_ET_) and ET rate (*k*_ET_) for the QD/PS hybrid structure assuming the relationships *η*_ET_ = 1 − (*τ*_QD/PS_/*τ*_bare_) and *k*_ET_ = *τ*_ET_^−1^ = *τ*_QD/PS_^−1^ − *τ*_bare_^−1^, where *τ*_bare_ and *τ*_QD/PS_ are the decay times for the bare QDs and QD/PS hybrid structure, respectively, and *τ*_ET_ is the characteristic time for the ET process^31^. The estimated *η*_ET_ and *k*_ET_ as a function of the *d*_QD_ are shown in Fig. 3(d). We determined a value of ~63% for the *η*_ET_ of the QD/PS hybrid structure with a *d*_QD_ of 2.7 nm. As the *d*_QD_ increased, the *η*_ET_ and *k*_ET_ decreased due to the decline in the driving force, which predominantly depends on the difference between the conduction band energies of the QDs and PS^32^. The band gap energy of the QDs decreased as the *d*_QD_ increased, which led to a reduced ET rate from the QDs to the PS, due to the smaller energetic offset between the CBM and VBM of the QDs and PS, as well as reduced overlap between emission spectrum of QDs and absorption spectrum of PS, as shown in Figs. 2 and S2(b), respectively. Therefore, the highest values of *η*_ET_ and *k*_ET_ observed for *d*_QD_ of 2.7 nm was consistent with the largest PL enhancement noted for the QD/PS hybrid structure with *d*_QD_ of 2.7 nm, as shown in Fig. 1(c).

**Figure 4:**
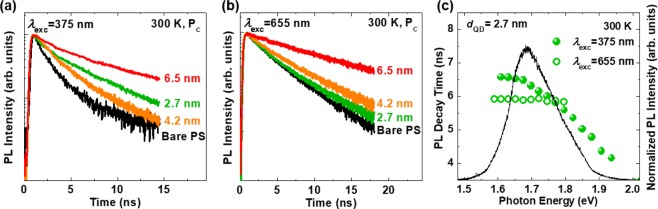
PL decay curves acquired at P_C_ for the bare PS and the QD/PS hybrid structures at 300 K using *λ*_exc_ of (**a**) 375 nm and (**b**) 655 nm. (**c**) Emission-photon-energy-dependent PL decay times of the QD/PS hybrid structure with *d*_QD_ of 2.7 nm excited by *λ*_exc_ of 655 nm (open circles) and 375 nm (filled circles). Additionally, the PL spectrum obtained at 300 K is shown.

Figure 4(a) displays the PL decay curves obtained at P_C_ for the bare PS and the hybrid structure using *λ*_exc_ of 375 nm at 300 K. The PL decay curves were adequately fitted by the bi-exponential decay function. The detailed decay parameters and average decay times are listed in Table 1. *τ*_ave_ recorded at P_C_ for the bare PS was 1.90 ns, and *τ*_ave_ for the QD/PS hybrid structure with *d*_QD_ of 2.7, 4.2, and 6.5 nm were 6.38, 4.37, and 9.19 ns, respectively. The decay time at P_C_ for the QD/PS hybrid structure was longer than that for the bare PS, which was attributed to ET from the QDs to the PS layer and reduced charge traps. Contrary to our expectation that the decay time would decrease as *d*_QD_ increases from 2.7 to 6.5 nm, the QD/PS hybrid structure with *d*_QD_ of 6.5 nm exhibited a considerably longer decay time. This result can be ascribed to the increased spatial separation of electrons and holes, owing to the electrons transferring to the PS while the holes move to the QDs, as shown in Fig. 2(c).

**Table 1:** Estimated PL decay parameters and average PL decay times taken at P_C_ for the bare PS and the QD/PS hybrid structures using a *λ*_exc_ of 375 nm at 300 K.

*d*_QD_ (nm)	*τ*_1_ (ns)	*τ*_2_ (ns)	*A*_1_ (%)	*A*_2_ (%)	*τ*_ave_ (ns)
0 (bare)	0.82	2.57	66.4	33.6	1.90
2.7	1.21	6.48	9.30	90.7	6.38
4.2	1.95	4.52	12.6	87.4	4.37
6.5	1.87	9.34	9.00	91.0	9.19

The TRPL spectra of the samples excited by *λ*_exc_ of 655 nm were obtained to exclude the excitation of QDs, as shown in Fig. 4(b). The PL decay curves of all samples were fitted adequately by a single exponential function. The estimated PL decay times are provided in the SI (Table S3). The PL decay time of the PS in the hybrid structure increased slightly from 3.07 to 3.40 ns as *d*_QD_ increased from 0 (bare PS) to 4.2 nm, which was attributed to the decreased number of trap states due to defect state passivation. The decay time of the hybrid structure with *d*_QD_ of 6.5 nm was longer than those of the other samples because *λ*_exc_ of 655 nm could partially excite the QDs, thus causing ET between the QDs and PS.

The emission-photon-energy-dependent PL decay curves for the QD/PS hybrid structure with *d*_QD_ of 2.7 nm excited by 655 nm and 375 nm *λ*_exc_ were obtained to investigate the dynamic nature of carriers in the QD/PS hybrid structure (see SI, Fig. S4). The estimated PL decay times for the QD/PS hybrid structure with *d*_QD_ of 2.7 nm excited by 655 nm and 375 nm *λ*_exc_ measured at 300 K are shown in Fig. 4(c) as a function of the emission photon energy. Further, the PL spectrum of the QD/PS hybrid structure (*d*_QD_ = 2.7 nm) measured at 300 K is shown. For the 655 nm excitation, which excited only the PS layer, the decay times were almost constant for the emission photon energies, while the decay times of the hybrid structure for the 375 nm excitation gradually increased from 4.17 to 6.57 ns as the emission photon energy decreased from 1.94 to 1.61 eV. This increase in the PL decay time at a low emission photon energy can be attributed to ET from the QDs to the PS layer.

## Conclusions

The luminescence properties of surface-passivated CH_3_NH_3_PbI_2_Br PS films covered with CdSe/ZnS core–shell QDs have been investigated using PL and TRPL measurements. The surface-passivated PS films (QD/PS hybrid structure) exhibited enhanced PL intensity and increased PL decay times compared with those of bare PS films. This observation is attributed to ET from the QDs to the PS and the reduced defect states resulting from QD surface passivation. The greatest enhancement in PL intensity for the QD/PS hybrid structure was obtained for a core diameter of 2.7 nm. The efficiency of ET from the QDs to the PS was determined to be ~63% for the core diameter of 2.7 nm. Our results facilitate an improved understanding of crucial fundamental and physical mechanisms, such as energy transfer between QDs and PS, for evaluating the effectiveness of surface passivation to improve film stability and reduce defects. Furthermore, the observation that the ET efficiency can be controlled by the size of the QDs can be potentially applied in the fabrication of PS-based optoelectronic devices with improved performance and stability.

## Methods

### Sample preparation

ITO treated glass substrates were ultrasonicated for 10 min in acetone and isopropyl alcohol, sequentially, and then exposed to UV-ozone treatment for 15 min at 100 °C. Approximately 120 mg of methylammonium bromide (Great Cell Solar Ltd.), 480 mg of lead iodide (Sigma Aldrich), and 1 ml of dimethyl sulfoxide (Sigma Aldrich) were dissolved in 2 ml of *N*,*N*-Dimethylformamide (Sigma Aldrich), and the solution of PS was mixed at 60 °C for 1 h^14^. The PS precursor solution was spin-coated by a two-step process on the ITO treated glass substrates: first at 1000 rpm (10 s) and then at 5000 rpm (20 s). During the second step, toluene (Sigma Aldrich) was dropped onto the film surface. Finally, the PS film was annealed for 2 min at 100 °C. 5 mg of CdSe/ZnS core-shell QD powder (PlasmaChem GmbH) was dissolved in 1 ml of toluene, and then the QD solution was mixed for 1 h at 60 °C. The CdSe/ZnS QDs precursor solution was then spin-coated at 3000 rpm (30 s) onto the PS film, and the QD/PS hybrid structure was finally annealed for 2 min at 60 °C.

### Measurement techniques

SEM (Zeiss Supra 55VP) and XRD (Malvern Panalytical X’pert Pro) measurements were performed to examine the surface morphology and crystallinity of the PS and the hybrid structures. UV-vis absorption spectra were collected by a Libra S80 spectrometer (Biochrom Ltd.). The PL signals were measured using a CCD detector (Andor DV420A-BU2) with a He-Cd laser (λ = 325 nm, Kimmon) as an excitation source. The TRPL spectra were collected using a microchannel-plate photomultiplier tube (FLS 920 spectrometer, Edinburgh Instrument). Picosecond pulsed diode lasers (excitation fluence = 0.1 µJ/cm^2^ and repetition rate = 20 MHz) were used as excitation sources for TRPL measurements, and the pulsed widths of 375 nm and 655 nm lasers were ~90 and 70 ps, respectively.

## Supplementary information

Supplementary information
