# Supplementary material for: Effect of energy transfer on the optical properties of surface-passivated perovskite films with CdSe/ZnS quantum dots
Source: Sci Rep. 2019 Dec 5;9:18433. doi: 10.1038/s41598-019-54860-1 (PMC6895127; doi:10.1038/s41598-019-54860-1)
Supplement: Supplementary file 1 — Supplementary information [file 41598_2019_54860_MOESM1_ESM.docx]

**Supplementary Information**

**Effect of energy transfer on the optical properties of surface-passivated perovskite with CdSe/ZnS quantum dots**

Il-Wook Cho and Mee-Yi Ryu

*Department of physics, Kangwon National University, Gangwon-Do 24341, Korea*

**1. Scanning electron microscopy (SEM) images of the bare PS and the QD/PS hybrid structure**

**
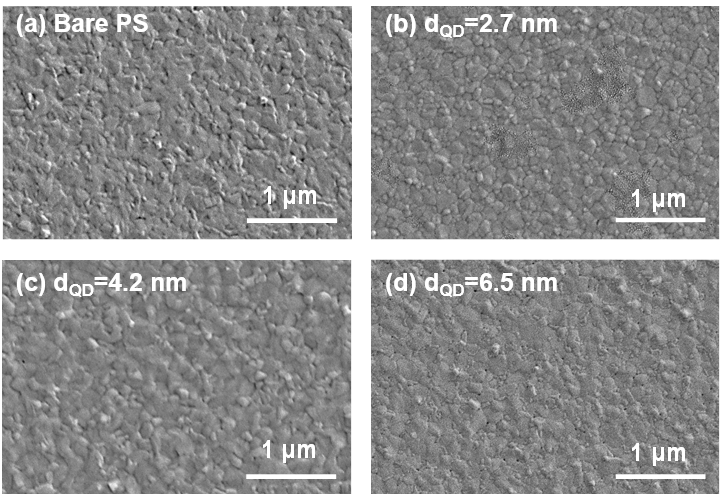
**

**Figure S1.** SEM images of (a) the bare PS and the QD/PS hybrid structures with *d*_QD_ of (b) 2.7 nm, (c) 4.2 nm, and (d) 6.5 nm. All SEM images show a similar grain size, indicating that the surface passivation with QDs did not significantly affect the morphology of CH_3_NH_3_PbI_2_Br film.

**2. X-ray diffraction (XRD) patterns and UV-vis absorption spectrum**

**
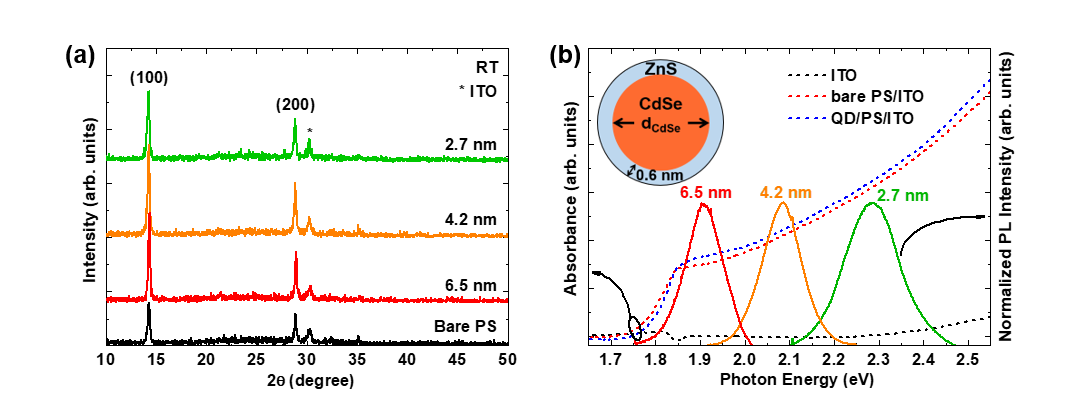
**

**Figure S2.** (a) XRD patterns of the bare PS and the QD/PS hybrid structures with various *d*_QD_. (b) UV-vis absorption spectrum of the ITO substrate, bare PS/ITO, and QD/PS/ITO (dashed lines) and PL spectra of the bare QDs with various *d*_QD_ (solid lines). The illustration of CdSe/ZnS core-shell QDs is shown as an inset.

**3. Estimated PL decay times of the bare QDs and the QD/PS hybrid structures**

**Table S1.** Estimated PL decay parameters and average PL decay times for bare QDs with varied *d*_QD_.

| *d*_QD_ (nm) | *τ*_1_ (ns) | *τ*_2_ (ns) | *A*_1_ (%) | *A*_2_ (%) | *τ*_ave_ (ns) |
| --- | --- | --- | --- | --- | --- |
| 2.7 | 1.29 | 4.59 | 60.6 | 39.4 | 3.59 |
| 4.2 | 1.32 | 4.27 | 59.8 | 40.2 | 3.34 |
| 6.5 | 0.78 | 2.99 | 56.3 | 43.7 | 2.44 |

**Table S2.** Estimated PL decay parameters and average PL decay times for QD/PS hybrid structures with varied *d*_QD_.

| *d*_QD_ (nm) | *τ*_1_ (ns) | *τ*_2_ (ns) | *A*_1_ (%) | *A*_2_ (%) | *τ*_ave_ (ns) |
| --- | --- | --- | --- | --- | --- |
| 2.7 | 0.63 | 2.50 | 86.8 | 13.2 | 1.33 |
| 4.2 | 0.65 | 3.18 | 83.8 | 16.2 | 1.88 |
| 6.5 | 1.30 | 2.79 | 53.1 | 46.9 | 2.27 |

**Table S3.** Estimated PL decay times taken at P_C_ for bare PS and QD/PS hybrid structures using *λ*_exc_ of 655 nm at 300 K.

| *d*_QD_ (nm) | 0 (bare) | 2.7 | 4.2 | 6.5 |
| --- | --- | --- | --- | --- |
| *τ* (ns) | 3.07 | 3.10 | 3.40 | 8.85 |

**4. PL decay curve and decay time of bare QDs in solution**

**
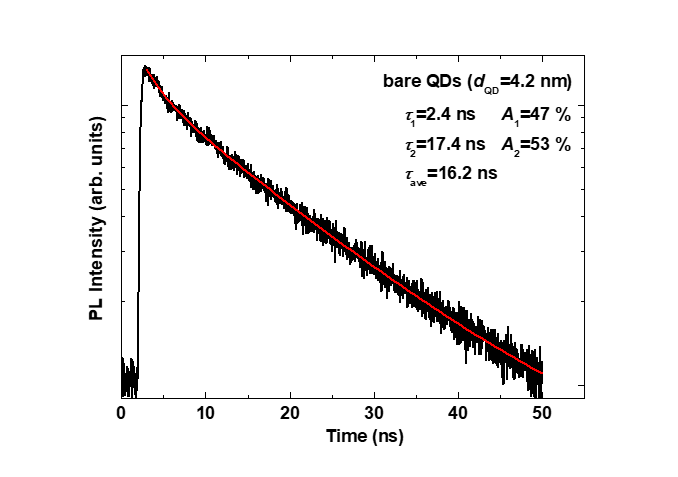
**

**Figure S3.** PL decay curve of the solution of bare QDs with *d*_QD_ of 4.2 nm measured at room temperature.

**5. Emission-photon-energy-dependent TRPL spectra of the QD/PS hybrid structure with *d*_QD_ of 2.7 nm**

**
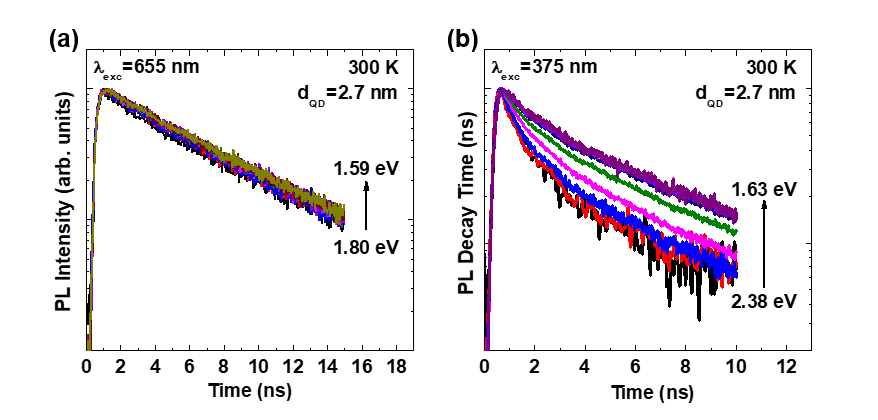
**

**Figure S4.** Emission-photon-energy-dependent PL decay curves of the QD/PS hybrid structure with *d*_QD_ of 2.7 nm by using (a) 655 nm and (b) 375 nm excitation sources measured at 300 K. For the 655 nm excitation that excites only the PS layer, the PL decay is nearly independent of emission photon energies. On the other hand, for the 375 nm excitation, which excites both the QDs and the PS, the PL decay increases with decreasing emission photon energy. The increase in the PL decay at low emission energy is attributed to the carrier transfer from QDs to PS layer.
